# The purpose of adaptation

**DOI:** 10.1098/rsfs.2017.0005

**Published:** 2017-08-18

**Authors:** Andy Gardner

**Affiliations:** School of Biology, University of St Andrews, Dyers Brae, St Andrews KY16 9TH, UK

**Keywords:** natural selection, fundamental theorem, Darwinism, inclusive fitness, social evolution, superorganism

## Abstract

A central feature of Darwin's theory of natural selection is that it explains the purpose of biological adaptation. Here, I: emphasize the scientific importance of understanding what adaptations are for, in terms of facilitating the derivation of empirically testable predictions; discuss the population genetical basis for Darwin's theory of the purpose of adaptation, with reference to Fisher's ‘fundamental theorem of natural selection'; and show that a deeper understanding of the purpose of adaptation is achieved in the context of social evolution, with reference to inclusive fitness and superorganisms.

## 1. The purpose of adaptation

Darwinism is a theory of the process of adaptation, i.e. the appearance of design in the biological world. The problem of how to explain adaptation is an ancient one, and it famously provided the basis for William Paley's [1] argument for the existence of an intelligent, divine designer. This problem was decisively solved by Charles Darwin [2], whose theory of evolution by natural selection—in which heritable variations associated with greater survival and reproductive success are identified as being more likely to accumulate in natural populations—explained the adaptation of organisms in purely naturalistic, mechanical terms.

Darwinism is also a theory of the purpose of adaptation, i.e. the design objective of biological organisms. Darwin argued that, as a consequence of natural selection preferentially retaining those heritable variations associated with greater survival and reproductive success, organisms will appear as if they are designed to maximize their survival and reproductive success—that is, their Darwinian fitness.

Indeed, Darwinism is the only scientific theory of the purpose of adaptation. While some continue to maintain that mystical forces—such as external, divine interventions or internal, vitalistic drives—are responsible for adaptation, none of these hypotheses yield clearly justified, testable predictions as to what the resulting adaptation is actually for (table 1). The question of purpose is often dodged, or else a purpose is asserted without clear justification. Strangely, whereas one might expect different drivers of adaptation to be associated with different design objectives, those who dispute natural selection's role in biological adaptation often nevertheless regard organisms as striving to maximize their Darwinian fitness. For example, anti-Darwinist James Shapiro [3, p. 137] views organisms as vitalistic beings that inexplicably strive to maximize their 'survival, growth and reproduction' for reasons that have nothing to do with the action of natural selection.

**Table 1.** Darwinism is the only scientific (i.e. predictive) theory of the purpose of adaptation.

	Darwinism	intelligent design, etc.
process	natural selection	divine intervention, etc.
purpose	maximize fitness	?

The idea of adaptive purpose does not imply that the design objective is perfectly realized. Paley [1] emphasized that the hallmark of design is not perfection but rather that an organism's or organ's apparent purposiveness is evident from its adaptive complexity, or ‘contrivance and relation of parts’. Comparing organisms and their component parts to human artefacts like pocket watches, he noted that even a broken watch manifests purposiveness in its intricate design. However, Paley—and Darwin after him—marvelled at how, in practice, nature abounds with exquisite adaptation that seems to border upon perfection.

In recognition of the distinction between purposefulness and perfection, it is useful to separate adaptationism into weak versus strong forms [4]. Weak adaptationism is the idea that organisms manifest apparent design and purpose, on account of the action of natural selection. Weak adaptationism makes no commitment to the idea of perfection, and recognizes that multiple forces in addition to natural selection—such as spontaneous mutation and random drift—contribute to the evolutionary process in an often deleterious way. By contrast, strong adaptationism is a caricature of Darwinism in which organisms are regarded as entirely optimal in their form and their behaviour. As a scientific hypothesis, strong adaptation is trivially falsified by empirical observation.

Yet, strong adaptationism is the central conceit of a hugely successful programme of scientific research, based upon optimization theory [5]. Practitioners of the optimization approach consider what organisms would be like if they *were* optimally fitted to the particular circumstances and challenges of their environment, and thereby derive predictions that although acknowledged to be only approximate are nevertheless, in practice, often useful ones. Indeed, when there is a marked discrepancy between prediction and empirical observation, this usually means that a key aspect of the organism's biology has not been properly understood and remains to be incorporated into the optimization model. Accordingly, by an iterative process of model adjustment, testable prediction and empirical test, the optimization approach provides an investigative tool by which scientists learn how the biological world works.

The optimization approach is made possible only because Darwinism yields such a clear prediction as to what biological adaptation is actually for. Without knowing what organisms are designed to do, it would be impossible to decide which of a range of possible phenotypes represents the optimum. This point clarifies why typical critiques of the adaptationist research programme are misguided: the perennial complaint that adaptationists fail to consider ‘other hypotheses’—for instance, that organisms may be to some degree maladapted [6]—mistakes adaptationism for a hypothesis when it is actually a research method. The maladaptation view is strictly correct but also completely useless if it does not yield specific, testable predictions. And it is detrimental to scientific progress if it obstructs the application of the successful adaptationist approach (cf. [7]).

## 2. The population genetics of purpose

The formal basis for evolutionary theory is the domain of theoretical population genetics. Accordingly, it is proper that Darwin's theory of the purpose of adaptation be framed in genetical terms. This was accomplished by Ronald Fisher [8,9], with what he termed the ‘fundamental theorem of natural selection' (box 1). Fisher's theorem provides a formal foundation for the view that natural selection leads organisms to maximize their fitness—in the sense that it will appear as if this is their purpose, rather than in the sense that they will necessarily perfectly realize this goal—and he rightly regarded it as taking centre stage in his masterpiece *The genetical theory of natural selection* [8]. But it has had a turbulent history.

Box 1. **Fundamental theorem of natural selection.**

*Price's equation*—In very general terms, evolutionary change can be expressed as a sum of selection and transmission components. This is captured by Price's [10,11] equation, based upon a general mapping between two populations of entities. Typically, one of these populations is descended from the other, and they are denoted 'parents' and 'offspring', respectively.

To derive Price's equation, assign every individual in the parent population a unique index *i* ∈ *I* and assign indices to every individual in the offspring population according to which parent individual they are descended from. When a given individual in the offspring population has more than one ancestor in the parent population (as in a sexual population), each ancestor is awarded its genetic share of the offspring. Denote the relative abundance of the *i*th parent as *q_i_*, where ∑*_I_ q_i_* = 1. Typically, *q_i_* = 1/*N*, where *N* is the number of individuals in the parent population. Similarly, denote the relative abundance of the *i*th parent's descendants in the offspring population as *q_i_*′. This allows a definition of the relative fitness of any individual in the parent population as *w_i_* = *q_i_*′/*q_i_*. Finally, assign each individual in the parent population a value *z_i_* for any character of interest, and denote the average character value of their offspring as *z_i_*′ = *z_i_* + Δ*z_i_*. The average character value over the parent and offspring populations is E(*z*) = ∑*_I_ q_i_ z_i_* and E(*z*′) = ∑*_I_ q_i_*′ *z_i_*′, respectively. Hence, the change in the population average value of the character of interest is ΔE(*z*) = E(*z*′) − E(*z*), which may be re-written as:B1.1
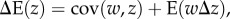

where: cov denotes a covariance and E an expectation, each taken over the set of all individuals in the population. The covariance term describes the change ascribed to the statistical association between an individual's character and its relative fitness, and defines *selection*. The expectation term describes the change ascribed to character differences between a parent and her offspring, and defines changes associated with *transmission*.

Natural selection is a particular type of selection that involves genes, the fundamental units of heredity. Here, the character of interest is not an individual's phenotype *per se*, but rather her (additive) genetic value for any phenotypic character of interest, i.e. the heritable portion of her phenotype [10,12]. Moreover, change is defined across a single generation. Denoting the genetic value by *g_i_*, the action of natural selection is given byB1.2
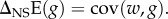

That is, the change in the average value of the heritable character ascribed to the action of natural selection is equal to the statistical covariance of that character and relative fitness, across all the individuals in the population. Importantly, equation (B 1.2) describes the action of natural selection only, and not the entirety of evolutionary change.

Without loss of generality, this may be re-written asB1.3
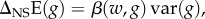

where var(*g*) is the heritable variance in the character of interest and *β*(*w*, *g*) = cov(*w*, *g*)/var(*g*) is the least-squares linear regression of relative fitness against the heritable character. This form of Price's equation highlights the basic Darwinian logic that natural selection will act to drive change in the heritable constitution of the population (Δ_NS_E(*g*) ≠ 0) if and only if there is heritable variation (var(*g*) > 0) in a character that is associated with individual fitness (*β*(*w*, *g*) ≠ 0).

*Fundamental theorem—*If the character of interest is taken to be fitness itself, then this may be decomposed into its genetical and environmental components, *w* = *g* + *e*. It follows that *β*(*w*, *g*) = 1, and substituting this into equation (B 1.3) obtains:B1.4
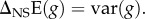

That is, the increase in average fitness ascribed to natural selection is equal to the genetic variance in fitness.

Fisher's clearest verbal statement of the fundamental theorem is: *the increase of average fitness of the population ascribable to natural selection is equal to the genetic variance of fitness*^1^ [9]. The salient point here is that, as variances are non-negative, there is a fundamental directionality to the action of natural selection, always pointing in the direction of increased fitness. That is, Fisher's theorem describes the optimizing quality of natural selection.

Despite Fisher's clear focus on the immediate action of natural selection, the fundamental theorem has long been interpreted as a statement about the total change in the population's fitness from one generation to the next. The idea that this would always increase was at first uncritically accepted and then, decades later, suddenly rejected when simple mathematical models revealed that population fitness is capable of decreasing from generation to generation [13]. This led to a widely held view that the fundamental theorem is not generally correct and—more damagingly—that any notion of fitness maximization, or of there being a clear purpose to Darwinian adaptation, is embarrassingly naive.

**Figure 1.**
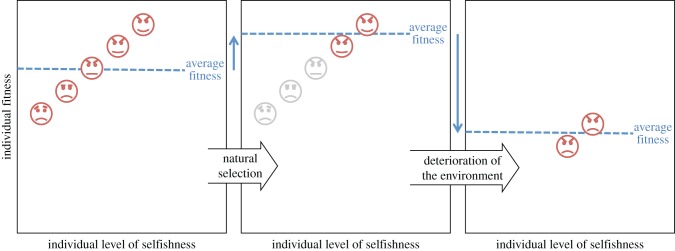
Change in average fitness ascribed to natural selection versus deterioration of the environment. In this example, each individual achieves a higher fitness if she behaves selfishly. Natural selection favours the fittest—i.e. most selfish—individuals, and the direct effect of this is to increase average fitness. However, the consequent deterioration of the social environment—owing to an increased average level of selfishness—leads individuals of all genotypes to have reduced fitness. The net effect is that average fitness decreases from one generation to the next.

With regard to its correctness, George Price's [14] careful exposition of Fisher's derivation established that the fundamental theorem is indeed mathematically sound (box 1). Price clarified that the fundamental theorem concerns only the part of change in average fitness across the individuals in the population that is due to the action of natural selection *per se* and not to other, non-Darwinian changes that Fisher [8] referred to collectively as *deterioration of the environment*. Figure 1 provides an illustration of which parts of the evolutionary change in average fitness are ascribed by Fisher to the action of natural selection versus environmental deterioration.

Price admitted to being disappointed that this partial result ‘does not say more'—presumably feeling that a description of the entirety of evolutionary change in population fitness would be preferable. However, it is precisely because the fundamental theorem is a partial result that it is so important [15]. In isolating the part of the evolutionary process responsible for adaptation—that is, natural selection—the fundamental theorem illuminates what is being adapted (the individual) and for what purpose (maximizing her fitness). Those individuals who achieve higher fitness are those whose heritable constitutions will predominate in future generations, and accordingly it is these individuals who point out the direction of the population's evolutionary future.

For example, in the scenario depicted in figure 1, individuals vary in their level of selfishness, with relatively selfish individuals having relatively higher fitness and relatively selfless individuals having relatively lower fitness, in comparison with their peers. Accordingly, the fitness-maximizing quality of natural selection leads to an increase in selfishness—as this is what directly increases the individual's fitness. (A secondary consequence is that all genotypes suffer reduced fitness on account of their carriers' social partners now having a greater tendency to behave selfishly, and this deterioration in the social environment results in a net decrease in average fitness.) That is, the idea that individuals strive to maximize their fitness correctly predicts the direction of evolutionary change.

It is not clear why a mathematical account of the total change in population fitness would be of much interest anyway. The relationship between population composition and population fitness does not point out the direction of the future. For example, in the above scenario, a population in which selflessness predominates enjoys greater fitness, but evolutionary change proceeds in the exact opposite direction: increased selfishness. This recovers the population geneticists' discovery that population fitness is not maximized [13], and makes clear that what they had rejected was not—as they had supposed—the idea that fitness is maximized, but rather the idea that adaptation works ‘for the good of the species’.

## 3. The purpose of social adaptation

The personal-fitness-maximization design principle emerging from Fisher's fundamental theorem is not a fully general result. It may fail when social interactions occur between genetic relatives. The application of the fundamental theorem to social evolution serves to underline how Fisher intended the theorem to be understood and yields deeper insights into the purpose of adaptation.

**Figure 2.**
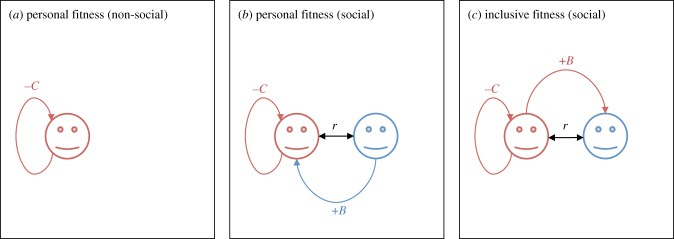
Personal fitness and inclusive fitness. (*a*) In the absence of social interaction between genetic relatives, the correlation between an individual's genotype and her personal fitness reflects the direct causal impact of her genotype on her personal fitness (−*C*). (*b*) In the context of social interaction between genetic relatives, the correlation between an individual's genotype and her personal fitness reflects the direct causal impact of her genotype on her personal fitness (−*C*) plus the correlation between her genotype and her social partner's genotype (*r*) multiplied by the causal impact of her social partner's genotype on her own personal fitness (+*B*). (*c*) In the context of social interaction between genetic relatives, the correlation between an individual's genotype and her inclusive fitness reflects the direct causal impact of her genotype on her personal fitness (−*C*) plus the causal impact of her genotype on her social partner's personal fitness (+*B*) multiplied by the relatedness valuation (*r*) she places upon her social partner's fitness.

In the absence of social interaction between genetic relatives—including, for example, the model of selfishness presented above—any correlation between an individual's genotype and her fitness may be taken to reflect a direct, causal relationship^2^ (figure 2*a*). That is, if an individual's heritable constitution is associated with higher fitness, this is because it actually increases her fitness. Accordingly, on account of natural selection favouring those traits that are associated with higher fitness, individuals will appear designed to maximize their fitness.

By contrast, if genetic relatives interact, then any correlation between an individual's genotype and her fitness may instead be due to her genotype being correlated with that of her social partner, and her social partner's genotype modulating her own fitness (figure 2*b*). That is, an individual's heritable constitution might be associated with higher fitness---and hence favoured by natural selection---even if it actually directly decreases her fitness. Accordingly, natural selection need not lead the individual to appear designed to maximize her personal fitness.

To be clear, social interaction between genetic relatives does not invalidate the fundamental theorem (the increase of average fitness of the population ascribable to natural selection is equal to the genetic variance of fitness, irrespective of social interaction between relatives; box 1). It merely prevents the fitness-maximization design interpretation from being drawn. So it is revealing that Fisher felt it necessary to assume the absence of social interaction between genetic relatives^3^ in his prelude to the fundamental theorem. That Fisher made this assumption indicates that he, too, drew the fitness-maximization design interpretation from his theorem.

Does this correlation–causation difficulty mean that natural selection is not responsible for organismal design in the context of social interaction between genetic relatives? Fortunately, this is not the case. The fundamental theorem may be reformulated using an alternative fitness measure—*inclusive fitness—*which is defined by subtracting from the individual's personal fitness all fitness effects due to the actions of her social partners, and adding all the fitness effects experienced by the focal individual's social partners as a consequence of her own actions, each increment or decrement being weighted by the focal individual's genetic relatedness to the corresponding recipient (box 2; figure 2*c*; [19]). That is, the fundamental theorem may be alternatively expressed as: *the change in average inclusive fitness ascribed to the action of natural selection is equal to the genetic variance in inclusive fitness* (box 2; cf. [20]).

Box 2. **Kin selection and inclusive fitness.**

*Kin selection*—On the assumption that there is heritable variation in a focal character (var(*g*) > 0) then, from box 1 equation (B 1.3), natural selection will act to increase the average value of this character (Δ_NS_*E*(*g*) > 0) if and only if the character is positively associated with individual fitness (*β*(*w*, *g*) > 0). There are two ways for a heritable character to be associated with greater personal fitness: first, the character may directly improve the individual's fitness (direct fitness benefit); and, second, the character may be present among the individual's social partners, such that its expression increases the individual's fitness (indirect fitness benefit). Using the mathematics of multiple least-squares regression, this may be expressed as:B2.1
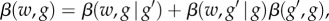

where *β*(*w*, *g* | *g*′) = −*C* is the effect of the individual's own heritable character *g* upon her own fitness *w*, holding fixed the heritable character of her social partner *g*′; *β*(*w*, *g*′ | *g*) = *B* is the effect of the individual's social partner's heritable character *g*′ upon her own fitness *w*, holding fixed her own heritable character *g*; and *β*(*g*′, *g*) = *r* is the statistical association between these social partners' heritable characters (i.e. the kin-selection coefficient of genetic relatedness; [18]). For simplicity, equation (B 2.1) assumes that the focal individual has only a single social partner, but the approach readily extends to scenarios in which the focal individual has multiple social partners.

Accordingly, the condition for natural selection to favour an increase in the average value of the heritable character (*β*(*w*, *g*) > 0) is −*C* + *Br* > 0, i.e. Hamilton's [19] rule of kin selection, expressed here in its personal fitness (or ‘neighbour-modulated fitness’) form.

*Inclusive fitness—*Noting that the statistical aggregate impact of social partners on the fitness of individuals within a population is identical to the statistical aggregate impact of individuals upon their social partners in that population (i.e. *β*(*w*, *g*′ | *g*) = *β*(*w*′, *g* | *g*′), where *w*′ denotes the relative fitness of an individual's social partner), the kin selection partition of natural selection into its direct and indirect components may alternatively be expressed in its inclusive fitness form:B2.2
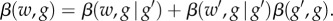

With some algebra, the action of natural selection can be expressed as:B2.3
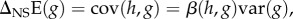

where *h* = *β*(*w*, *g* | *g*′)*g* + *β*(*w*′, *g* | *g*′)*β*(*g*′, *g*)*g* is the focal individual's inclusive fitness, i.e. the sum of her heritable character's impact on her personal fitness and also on the personal fitness of her social partner, the latter being weighted by the degree of genetic relatedness between the two parties [19].

*Fundamental theorem*—If the character of interest is taken to be inclusive fitness itself, then this may be decomposed into its genetical and environmental components, *h* = *g* + *e*. It follows that *β*(*h*, *g*) = 1, and substituting this into equation (B 2.3) obtainsB2.4
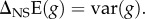

That is, the increase in average inclusive fitness ascribed to natural selection is equal to the genetic variance in inclusive fitness (cf. [20]).

By virtue of its definition, inclusive fitness is under the individual's full control, such that the correlation between an individual's genotype and her inclusive fitness reflects a direct causal relationship. Accordingly, as a consequence of the action of natural selection the individual appears designed to maximize her inclusive fitness.

Though Darwinian adaptation is conventionally viewed as occurring at the level of the individual organism, recent years have seen growing interest in the idea that whole social groups may be viewed as ‘superorganisms’ in their own right, wielding their own adaptations for their own purposes. In some cases, this is simply a return to the woolly thinking of the first half of the twentieth century, when many biologists unreflectively regarded natural selection as always working for the good of the group or species. However, in other cases, there is a legitimate recognition that—on rare, but important, occasions—groups of socially interacting individuals have undergone a major transition in individuality, such as the transition from unicellular to multicellular life, and from cooperative breeding to eusociality [21,22].

The fundamental theorem approach may be brought to bear on this question of group-level adaptation. Specifically, the action of natural selection may be decomposed into the component operating at the within-group level and the component operating at the between-group level, and in taking group fitness itself to be the character of focal interest a fundamental theorem of multi-level selection emerges that states: *the change in average group fitness owing to the action of natural selection is equal to the genetic variance in group fitness if and only if there is no selection within groups* (box 3; [24]). This result clarifies why certain animal groups—like the Portuguese man-of-war, a jellyfish-like colony of clonally related zooids, within which there is essentially no genetic variation and hence no scope for within-colony selection—can be considered adapted superorganisms in their own right, but most animal groups—within which there is scope for conflict as well as collaboration—cannot.

Box 3. **Fundamental theorem of multi-level selection.**

*Multi-level selection—*In a group-structured population, the action of natural selection may alternatively be separated into its between-group and within-group components:B3.1
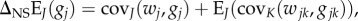

where I have assigned every group a unique index *j* ∈ *J*; and, within a given group, I have assigned every individual a unique index *k* ∈ *K*. The first term on the r.h.s. defines between-group selection and the second term on the r.h.s. defines within-group selection [11,23].

*Fundamental theorem—*If the character of interest is taken to be group fitness itself, then this may be decomposed into its genetical and environmental components, *w_j_* = *g_j_* + *e_j_*. It follows that *β_J_*(*w_j_*, *g_j_*) = 1, and hence cov*_J_*(*w_j_*, *g_j_*) = *β_J_*(*w_j_*, *g_j_*) var*_J_*(*g_j_*) = var*_J_*(*g_j_*), and substituting this into equation (B 3.1) obtainsB3.2
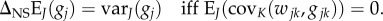

That is, the increase in average group fitness ascribed to natural selection is equal to the genetic variance in group fitness if and only if within-group selection is absent [24].

## 4. Conclusion

Explaining the purpose of adaptation is a central achievement of Darwinism. Being able to predict what it is that organisms are striving to achieve not only sets Darwinism apart from intelligent design and other forms of mysticism, but also sets the hugely successful adaptationist research programme apart from scientifically sterile anti-adaptationist thinking within evolutionary biology. Getting to grips with the purpose of adaptation is especially important in the context of social evolution, where different biological agents are expected to have different, conflicting purposes, and where naive notions of group-level adaptation are liable to be strongly misleading. Happily, there is a maturing body of formal theory that equips the evolutionary biologist with the tools required to navigate these issues and to break new Darwinian ground.

## Endnotes

1 In full: ‘The increase of average fitness of the population ascribable to a change in gene frequency *dp* will be 2*αdp*. Hence the rate of increase in the average value of the Malthusian parameter ascribable to natural selection acting on a single factor is 2*pqaα*, and the rate of increase due to all factors will be 2∑*pqaα*, equal to the genetic variance of fitness due to all factors’ [9].

2 Spurious correlations between genotype and fitness may be classified as owing to chance (in which case, they may be removed by defining fitness in terms of an expectation taken over uncertainty; [16]) or to class (which is explicitly controlled for in Fisher's [8] treatment, but neglected here for simplicity; see also Price & Smith [17]).

3 ‘There will also, no doubt, be indirect effects in cases in which an animal favours or impedes the survival or reproduction of its relatives; as a suckling mother assists the survival of her child, as in mankind a mother past bearing may greatly promote the reproduction of her children, as a foetus and in less measure a sucking child inhibits conception, and most strikingly of all as in the services of neuter insects to their queen. Nevertheless such indirect effects will in very many cases be unimportant compared to the effects of personal reproduction’ [8, p. 27].

## Data accessibility

This article has no additional data.
